# The Application of a Fluoride-and-Vitamin D Solution to Deciduous Teeth Promotes Formation of Persistent Mineral Crystals: A Morphological Ex-Vivo Study

**DOI:** 10.3390/ma16114049

**Published:** 2023-05-29

**Authors:** Gianni Di Giorgio, Michela Relucenti, Flavia Iaculli, Alessandro Salucci, Orlando Donfrancesco, Antonella Polimeni, Maurizio Bossù

**Affiliations:** 1 Department of Oral and Maxillofacial Sciences, Sapienza University of Rome, 00161 Rome, Italy; gianni.digiorgio@uniroma1.it (G.D.G.); alessandro.salucci@uniroma1.it (A.S.); antonella.polimeni@uniroma1.it (A.P.); maurizio.bossu@uniroma1.it (M.B.); 2 Department of Anatomy, Histology, Forensic Medicine and Orthopaedics, Sapienza University of Rome, 00161 Rome, Italy; michela.relucenti@uniroma1.it (M.R.); orlando.donfrancesco@uniroma1.it (O.D.); 3 Department of Neuroscience, Reproductive Sciences and Dentistry, University of Naples Federico II, 80131 Naples, Italy

**Keywords:** fluoride, dental caries, preventive dentistry, primary teeth enamel, remineralization, surface analysis, vitamin D, VPSEM

## Abstract

*Background*: The use of effective, low-cost, and easy-to-use products for early caries management will avoid loss of dental vitality and impairment in oral function. The ability of fluoride to re-mineralize dental surfaces has been widely reported as well as vitamin D demonstrated to have significant potential in improving the remineralization of early lesions on enamel surfaces. The aim of the present ex vivo study was to evaluate the effect of a fluoride and vitamin D solution in terms of formation of mineral crystals on the enamel of primary teeth, and their permanence over time on dental surfaces. *Methods*: Sixteen extracted deciduous teeth were cut to obtain 64 specimens that were divided into two groups. The first consisted of immersion of specimens for 4 days in a fluoride solution (T1); in the second group, the specimens were immersed for 4 days (T1) in fluoride and Vitamin D solution, and for a further 2 (T2) and 4 days (T3) in saline solution. Then, samples were morphologically analyzed by using Variable Pressure Scanning Electron Microscope (VPSEM) and underwent 3D surface reconstruction. *Results*: After a 4-day immersion in both solutions, octahedral-shaped crystals were formed on the enamel surface of primary teeth, demonstrating any statistically significant differences in terms of number, size, and shape. Moreover, the binding of the same crystals seemed to be strong enough to be maintained until 4 days in saline solution. However, a partial dissolution was observed in a time-dependent manner. *Conclusions*: A topical application of fluoride and Vitamin D promoted the formation of persistent mineral crystals on enamel surfaces of deciduous teeth and should be further studied to be potentially used as an alternative strategy in preventive dentistry.

## 1. Introduction

Dental caries is an infective, chronic, degenerative, and multifactorial condition that represents the most prevalent chronic disease worldwide, mainly in children [1,2]. The Global Burden of Disease Study 2019 estimated that oral diseases affect close to 3.5-billion people worldwide, with caries of permanent teeth being the most common condition [3]. Specifically, it has been estimated that 2-billion people suffer from caries of permanent teeth and 520-million children are affected by caries of primary teeth [3]. Despite the preventive strategies mostly adopted in developed countries, in most low- and middle-income areas, the prevalence of oral diseases is still increasing due to expanding urbanization and variation in living conditions. This is mainly caused by inadequate prevention, the availability of food with high sugar content, and limited exposure to oral health national care services [3].

Early caries management, both in deciduous and permeant dentition, should avoid the progressive destruction of dental hard tissue, subsequent loss of dental vitality, and impairment in oral function [4]. This is mostly true for primary teeth—due to anatomical considerations, reduced rate of mineralization, and high prevalence of risk factors—that show a rapid progression of dental caries [2,5]. Human teeth are constantly subjected to a continuous process of de- and re-mineralization, mostly due to pH alteration, cariogenic bacteria, external agents, heat, cold, and sweets, as well as erosive food and drink. The main issue concerning demineralization is that the loss of mineralized dental tissues favors the development of demineralized areas that may evolve in dental caries. This aspect is most prevalent in primary teeth since the enamel appears to be thinner, less mineralized (mineralization of 80.6% in deciduous enamel and 89.7% in permanent enameled teeth), and with not completely formed prisms at the outer layer [6].

The ability of fluoride to re-mineralize dental surfaces has been widely demonstrated within the scientific literature for both permanent and deciduous teeth [7]. Fluoride ions are able to be incorporated within tooth enamel during the dental development phase and, at adequate concentrations, support mineralization and decrease enamel solubility; the resulting fluorapatite on tooth surfaces had been demonstrated to be more resistant to acid attack [8]. Although excessive fluoride assumption may lead to dental fluorosis of permanent teeth, newly developed biomimetic toothpastes have been proposed as valid alternatives to minimize the risk of fluorosis occurrence, providing promising results in preventive dentistry [9,10]. In addition, in cases of poor oral hygiene abilities as well as limited contact between remineralized agents and dental surfaces, different fluoride delivery systems, such as sprays, have been introduced.

Vitamin D, among other functions such as innate immunity effector, plays an important role in the regulation of calcium and phosphate metabolism and their deposition in mineralized tissues [11]. Since the presence of Vitamin D3 receptors on ameloblast and odontoblast cells [12], its function in tooth development and remineralization, changes in the biochemical composition of saliva and immunological modulation of dental infection [13] has been suggested. Moreover, its deficiency has been reported as a risk factor both for the incidence of dental caries and their severity in children [14]. Indeed, Vitamin D has been demonstrated to have significant potential in improving the remineralization of early lesions on enamel surfaces enhancing surface microhardness and minerals content [15], suggesting a topical fluoride-like effect [13].

However, to the best of our knowledge, there are limited data about the effectiveness of fluoride and Vitamin D association in terms of dental caries prevention [16]. Therefore, the aim of the present study was to ex vivo prove the effect of fluoride and Vitamin D association on the enamel surface of human deciduous teeth in terms of mineral crystal formation. In addition, further studies are already planned to test the permanence, deepening of penetration, and remineralization effect of the same solution in an environment that simulates the oral cavity. Finally, a subsequent in-vivo study will elucidate the effectiveness of fluoride and Vitamin D association on enamel surface remineralization of human primary teeth and its role in preventive dentistry.

## 2. Materials and Methods

### 2.1. Samples Selection and Cutting Procedure

**Figure 1:**
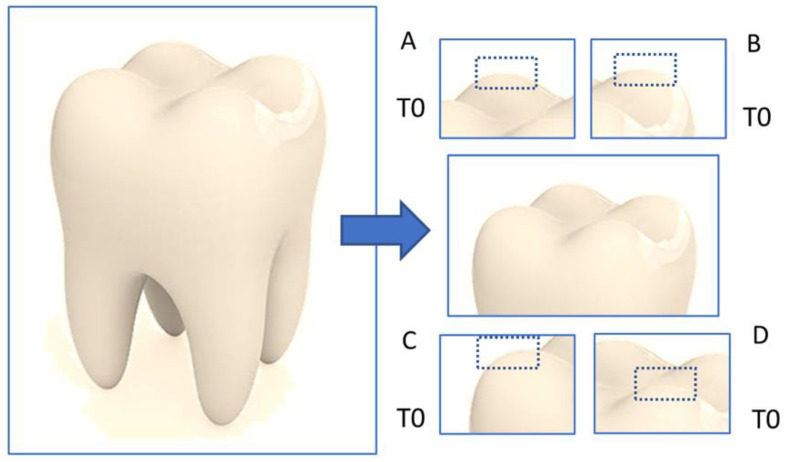
Samples’ cutting procedure to obtain 4 parallelepipeds of 4 mm × 4 mm (**A**–**D**) from one tooth; the native state was named T0. The dotted rectangle indicates the observed area in each sample (the apex of the cuspid) to avoid methodological biases.

A total of 16 deciduous teeth were extracted for orthodontic reasons or due to physiological replacements at the Pediatric Dentistry Unit of “Sapienza” University of Rome. Parents of all subjects signed an informed consent during the first visit, allowing for the use of extracted teeth for research purposes. The study was conducted under the Declaration of Helsinki, and the protocol was approved by the Ethical Committee of “Sapienza” University of Rome (Prot. 0885/2021 del 14/10/2021). After the exclusion of teeth reporting cracks or defects, specimens were cut with a diamond disk under water-spray irrigation, as previously reported [17]. To create samples with standard dimensions, the crowns of the 16 deciduous teeth were cut forming 64 parallelepipeds of size 4 mm × 4 mm (Figure 1). Then, the 64 specimens were analyzed through an optical microscope (Zeiss, Jena, Germany, Axioskop 40) at 20× magnification. All experimental procedures were performed by the same operator in the same conditions.

### 2.2. Experimental Design

**Figure 2:**
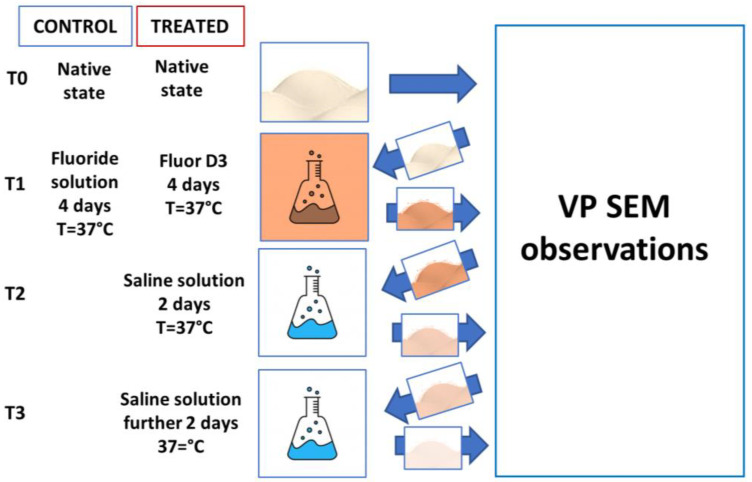
Representative image of study design. Immediate observation was allowed using Variable Pressure Scanning Electron Microscope (VP–SEM), which did not need sputter coating.

The specimens were divided into two groups of 32 samples each. The first group was used as a control and was processed as follows: (a) Observation just after the cutting procedure (T0); (b) Immersion for 4 days in a fluoride solution of 2.625 mg/mL in an oven at 37 °C, then under Variable Pressure Scanning Electron Microscope (VP– SEM) observation (T1). The second group (treated) was processed as follows: (a) Observation just after the cutting (T0); (b) Immersion over 4 days in a solution mainly containing fluoride (same concentration of control group) and Vitamin D3 (FluorD3, Dicofarm, Rome, Italy) in an oven at 37 °C (T1), and then under VP– SEM observation; (c) Immersion in saline solution (sodium chloride 0.9%) for 2 days in an oven at 37 °C, and then under VP– SEM observation (T2); (d) Additional 2 days immersion in saline solution (sodium chloride 0.9%) in an oven at 37 °C, and then under VP– SEM observation (T3) (Figure 2).

### 2.3. Sample Preparation for Variable Pressure Scanning Electron Microscope (VP–SEM)

Samples were observed without any drying or coating procedure. Specimens were carefully dried at room temperature by resting on a filter paper for 1–2 min. Then, they were mounted onto aluminum stubs by carbon tape and placed in an oven at 37 °C for 2 h before observation with the variable pressure SEM Hitachi SU–3500 (Hitachi, Tokyo, Japan). The observations were performed at operating conditions of 30 Pa and 5 to 10 kV. Images were captured at several magnifications between 500 and 5000×.

### 2.4. 3D Reconstruction and Surface Analysis Procedure

Crystals surface measurements and 3D reconstructions were carried out on pictures captured at 2000× and 5000× magnification with the aid of Hitachi 3d Map software (Digital Surf, Beçason, France) [18]. Data were analyzed by MedCalc© statistical software Version 22.003 (MedCalc©, Ostende, Belgium).

### 2.5. Statistical Analysis

To determine the significant differences between crystals’ number and sizes in the control and treated groups, as well as crystals’ number between T1 and T2 and between T2 and T3, *t*-Test was conducted by MedCalc^®^ Statistical Software version 20.218 (MedCalc Software Ltd., Ostend, Belgium; https://www.medcalc.org; (accessed on 10 April 2023).

## 3. Results

**Figure 3:**
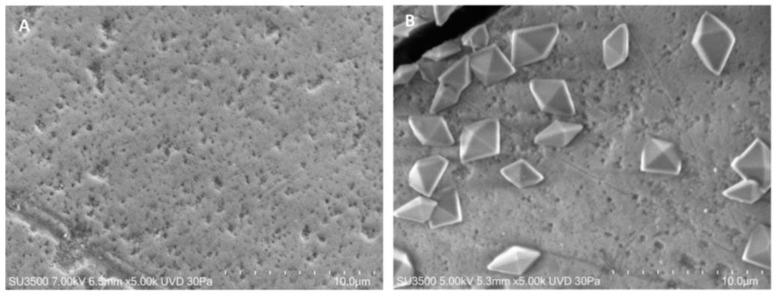
VPSEM observation. (**A**) Representative image of the surface of a sample without any treatment belonging to one of the two groups. The surface was marked by scratches and small holes; magnification 5.00 K. (**B**) Representative image of the surface of a sample after 4 days of immersion or in Fluoride solution or FluorD3 solution, numerous crystals with octahedral shape are visible; magnification 5.00 K.

The enamel surface at T0 in both control and treated groups showed furrows due to wear, shallow punctiform microcavities, and lacunae with larger surface areas and depths (Figure 3A). At T1, the enamel surface showed the formation of fluoride crystals with a characteristic octahedron shape within all evaluated samples (Figure 3B).

To verify the ability to form mineral crystals, their number in the control (fluoride solution) and treated group (FluorD3) was appreciated by observing 105 microscopic fields. Results are reported in Table 1.

**Figure 4:**
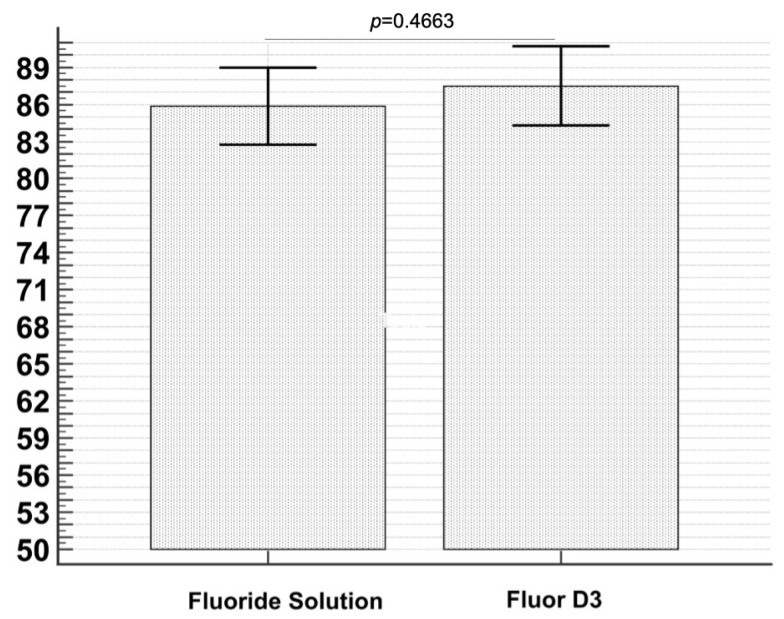
The graph illustrates the comparison between the number of crystals formed after immersion in control and test groups. No statistically significant differences were evidenced by the *t*-Test (*p =* 0.4663).

Then, the number of crystals formed after immersion for 4 days in control and test solutions was compared by a *t*-Test. The crystals number did not show any statistically significant difference (*t*-Test *p* = 0.4663) (Figure 4), suggesting that the presence of Vitamin D3 had no significant effect on crystals’ number formation.

**Figure 5:**
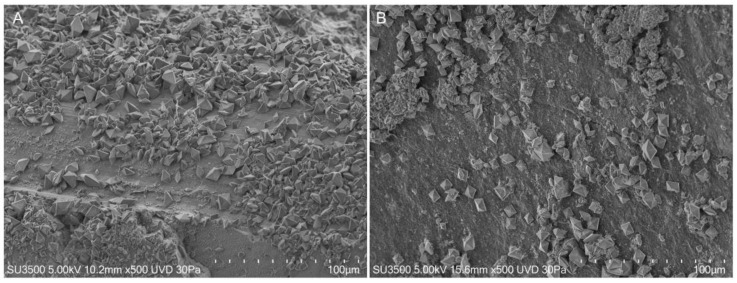
(**A**) Surface of a sample immersed in fluoride solution, 500×. Numerous crystals with octahedral shapes and different orientations are visible. (**B**) Surface of a sample immersed in fluoride and Vitamin D3 solution, 500×. The formed crystals have an octahedral shape.

The formed crystals’ shape was evaluated by panoramic views of the samples’ surfaces at low magnifications (Figure 5). The shape of all observed crystals in both groups was a typical octahedron.

Then, the crystals’ size was detected by the software Hitachi Mountains 3D, measuring the size of 150 crystals with the same orientation to avoid biases. Results are reported in Table 2.

**Figure 6:**
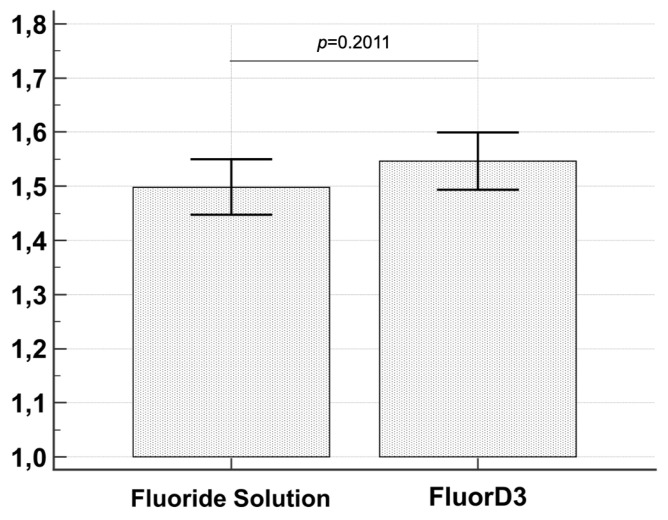
The graph illustrates the comparison between the size of crystals formed after immersion for 4 days in control and test solutions. No statistically significant differences were evidenced by the *t*-Test (*p =* 0.2011).

Comparison of crystals’ size did not show any statistically significant difference (*t*-Test *p* = 0.2011) (Figure 6).

**Figure 7:**
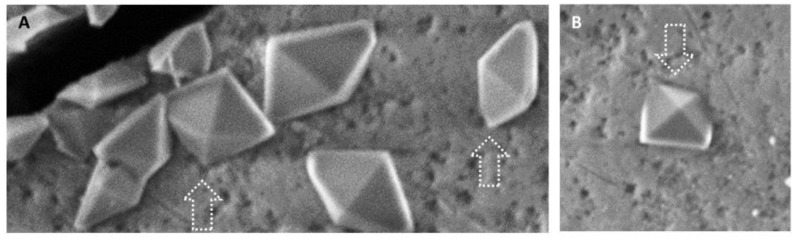
VPSEM observation. (**A**) Detail of Picture B showing crystals emerging with one vertex (arrow) from the enamel surface microcavities. (**B**) One crystal originates with two vertexes and one side (arrow) from the enamel surface irregularities.

Observing crystals at higher magnifications (Figure 7), they seemed to originate by crystallizing on the enamel surface microcavities, which serve as nucleation sites. The crystals then grew on the enamel surface in different orientations, some having a vertex in the microcavities, and the rest as free. In other cases, crystals grew by keeping their half anchored to the enamel (with two vertices and the side between them).

**Figure 8:**
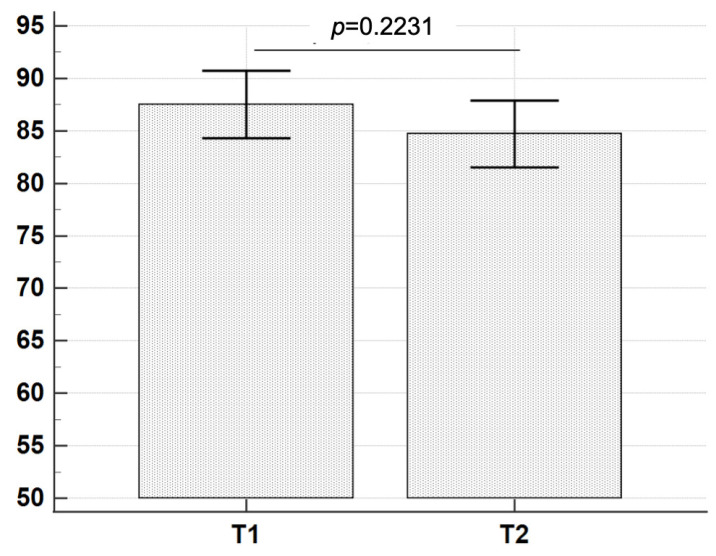
The graph illustrates the distribution of crystals’ number present in the same 105 microscopic fields in the same samples at T1 and T2. No statistically significant differences were evidenced by the *t*-Test between crystals’ number at T1 and T2 (*p* = 0.2231).

To test whether the binding of crystals to the enamel surface was strong enough to ensure their position over time, specimens were kept in a saline solution for 2 days and then observed. Analyzing the same 105 microscopic fields in the same samples at T1 and T2, the crystal number did not show any statistically significant difference (*t*-Test *p =* 0.2231) (Table 3, Figure 8). These results indicated that the bond between the crystal and enamel surface was sufficiently stable to keep the crystals adhering to the enamel surface after 2 days of immersion in saline solution.

**Figure 9:**
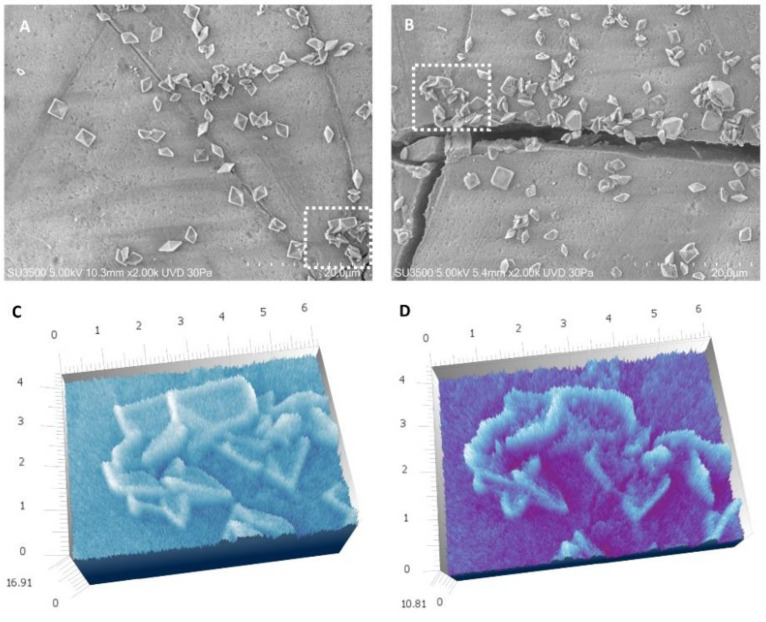
VPSEM observation and 3D reconstruction. (**A**) A microscopic field imaged at 2.00 K at T1. (**B**) The same field at T2. (**C**) 3D reconstruction of a dotted rectangle in T1. (**D**) 3D reconstruction of a dotted rectangle in T2. In Sample T2, the surfaces appeared rougher than in T1.

To evaluate crystals’ surface characteristics and size changes, and their total or partial dissolution, the size of 15 crystals present in the same microscopic fields at T1 and T2 was measured by Hitachi Mountains 3D software. The 3D reconstruction in Figure 9 shows that in T2, the size, shape, and roughness were different than that in T1, probably indicating an erosion process.

**Figure 10:**
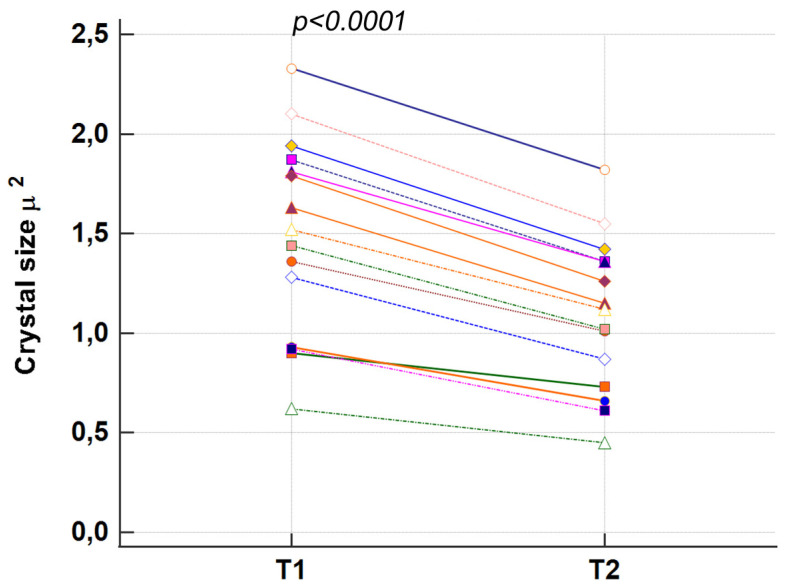
The graph illustrates the distribution of size values of the same 15 crystals at T1 and T2. Statistically significant differences were evidenced by the *t*-Test between crystal sizes at T1 and T2 (*p* < 0.0001).

To achieve a more precise quantitative evaluation, the size of each crystal was measured at T1 and T2, and a *t*-Test between the values was performed. Data showed that the size of every single crystal in T2 was inferior to the respective size in T1, and *t*-Test results demonstrated that this difference was statistically significant (*p* < 0.0001). Our data showed that the crystals have partially dissolved and lost an average percentage of 26% of their size after 2 days in saline solution (Table 4 and Figure 10).

**Figure 11:**
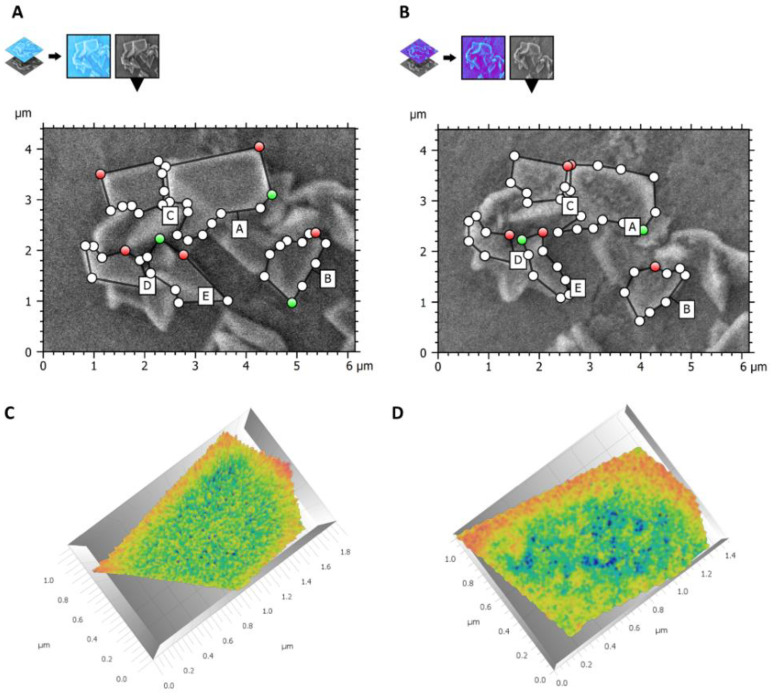
Surface analysis and 3D reconstruction. (**A**) Size measurement of crystals in the microscopic field imaged at 2.00 K at T1. (**B**) Size measurement of crystals in the same field at T2. (**C**) 3D surface reconstruction of T1 crystal in false colors (yellow: high level; green: middle level; blue: deep level). (**D**) 3D surface reconstruction of T2 crystal in false colors (yellow: high level; green: middle level; blue: deep level). The surface in T2 appeared porous and eroded, as shown by the presence of larger blue areas than T1.

The crystals’ 3D surface reconstruction in Figure 11 shows that at T2, the surface presented more areas of blue and dark green (lower-level areas) than T1, where yellow (high-level areas) and light-green areas (middle-level areas) prevailed. This meant that crystals had lower sizes and a different, more porous aspect.

**Figure 12:**
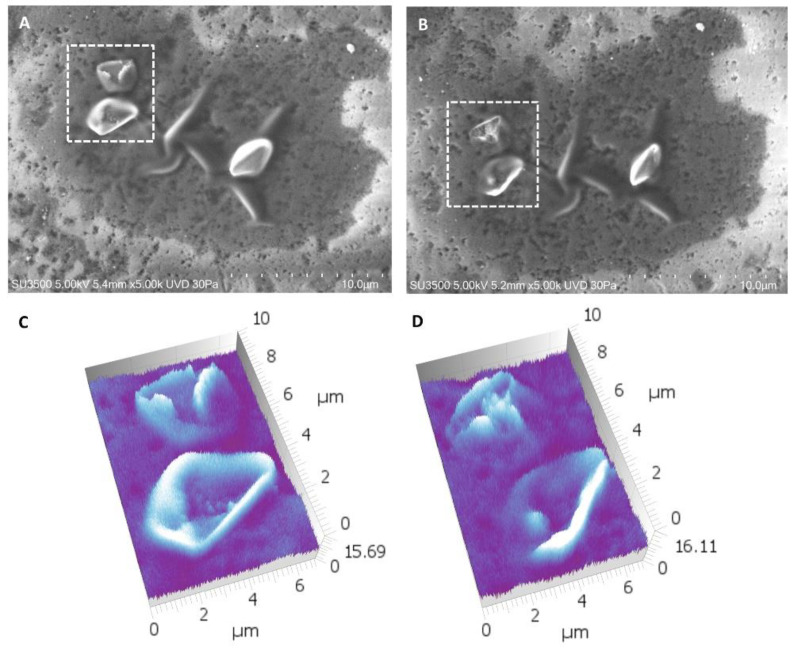
VPSEM observation. (**A**) A microscopic field imagined at 5.00 K at T2. (**B**) The same field at T3. (**C**) 3D reconstruction of the dotted rectangle at T2. (**D**) 3D reconstruction of the dotted rectangle at T3. The reconstruction emphasized that crystals were still in place even if deeply eroded.

To verify the trend of size decreasing and surface changes in the function of time, the size of 15 other crystals (different from the ones previously observed) at T2 and T3 was measured by using Hitachi Mountains 3D software. The 3D reconstruction in Figure 12 shows that in T3, the sizes, shape, and roughness were different than that in T2, stressing a further development of the erosion process.

**Figure 13:**
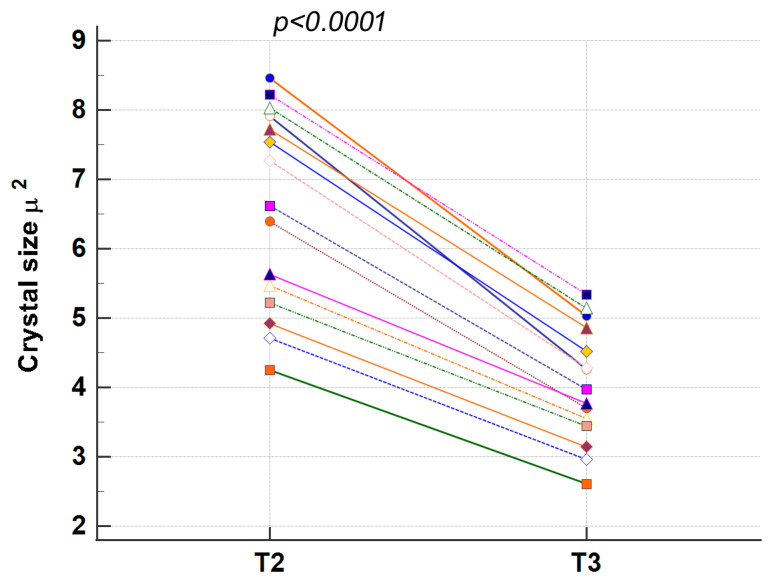
The graph illustrates the distribution of size values of the same 15 crystals at T2 and T3. Statistically significant differences were evidenced by the *t*-Test between crystals’ sizes at T2 and T3 (*p* < 0.0001).

To quantify the changes, the size of each crystal was measured at T2 and T3, and a *t*-Test between the values was performed. Data showed that the size of every single crystal in T3 was inferior to the respective size in T2, and the *t*-Test result demonstrated that this difference was statistically significant (*p* < 0.0001). Our data showed that the crystals had partially dissolved and lost an average percentage of 38% of their size after 4 days in saline solution (Table 5 and Figure 13).

**Figure 14:**
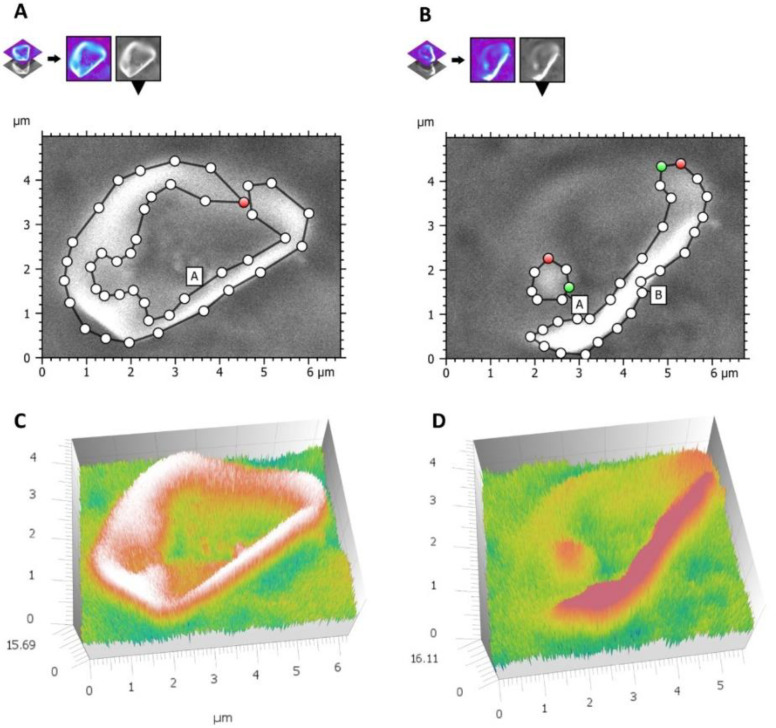
Surface analysis and 3D reconstruction. (**A**) Surface measurement of a crystal in the microscopic field imaged at 5.00 K at T2. (**B**) Surface measurement of crystal at the same field at T3. (**C**) 3D surface reconstruction of a crystal at T2 in false colors (white: highest level; red: middle level; green: ground level). (**D**) 3D surface reconstruction of the same crystal at T3 in false colors (white: highest level; red: middle level; green: ground level). The reduction of crystal surface in T3 was evident. showing the absence of white areas and an almost complete erosion of the perimeter.

The 3D surface reconstruction in Figure 14 shows that at T3, the crystal surface presented no white areas (high-level areas), whereas red and light-green areas (middle- and ground-level areas) prevailed. This meant that crystals had a marked lowering in size.

## 4. Discussion

Prevention strategies should be applied to reduce risk factors related to the onset and further development of dental caries, mainly in the early phase of childhood [19]. The preschool period occurs at a time in which deleterious oral habits, caries patterns, and risk factors are established, reporting a prevalence of around 30–80% [20]. In addition, limited oral health significantly impaired children’s nutrition and, in turn, general health and development, causing notable economic, public, and individual issues [21]. Childhood dental caries may be in relation to not only the children’s oral health-related quality of life, but also their parents’ [22]. To the best of our knowledge, this is the first study aimed at evaluating the behavior of fluoride and Vitamin D on enamel surfaces of human primary teeth. The present ultrastructural morphological analysis revealed that after a 4-day immersion of fluoride and Vitamin D solution, octahedral-shaped crystals were formed on the enamel surface without any statistically significant differences with a fluoride solution used as a control in terms of crystals’ number, shape, or size. Moreover, the binding of the same crystals on enamel seemed to be strong enough to be maintained until 4 days in a saline solution. However, a partial dissolution was appreciated in a time-dependent manner. Although the present study only provided an objective observation (the outcomes should be considered preliminary), the dissolution behavior over time might suggest that, in a clinical condition, the solution should be daily renewed to guarantee an efficient contact between the components and the enamel surface. In addition, following the hypothesis that the association of both fluoride and Vitamin D might be considered a valid alternative in preventive dentistry, their clinical use would be supported. Accordingly, Kühnisch et al. [16] reported promising results in terms of a lower probability of dental caries in primary dentition, as well as molar incisor hypomineralization (MIH) when children underwent Fluoride/Vitamin D tablet supplementation during the first year of life. In addition, the same authors observed no effects on permanent dentition 10 years after the administration, suggesting only the preventive role of fluoride and Vitamin D association with the absence of further damages [16]. As suggested by the present study, the topical application of the same components should overcome drawbacks related to an excessive assumption of fluoride, mainly in children of less than 5 years of age, who are more at risk of fluorosis occurrence due to the swallowing of a high amount of fluoride toothpaste during the mineralization phase of permanent teeth [23]. In addition, an easy topical delivery system might have a positive effect on the extension of early childhood caries prevention, as well as an economic impact on health care services.

The present study was based on the rationale that, alone, fluoride and Vitamin D positively act on enamel surface remineralization. However, according to the provided preliminary results, it only proved the presence and maintenance of mineral crystals on enamel surfaces. Moreover, although the use of saline solution was chosen to establish the crystal formation kinetics without the possible interference made by saliva and its components, its presence would have better reproduced the oral environment, representing a main limitation of the work. Therefore, further ex-vivo and in-vivo studies are already planned to demonstrate the deepening of penetration and the surface remineralization potential, and to show as a combination of topically administered fluoride and Vitamin D may be considered a valid alternative in preventive dentistry.

## 5. Conclusions

Within the limitations of the present study, it could be concluded that the topical application of fluoride and Vitamin D promoted the formation of persistent mineral crystals on enamel surfaces of deciduous teeth and should be further evaluated to be potentially used as an alternative strategy in preventive dentistry.

## Figures and Tables

**Table 1:** Summary statistics of crystals’ number formed after immersion for 4 days in fluoride solution and after immersion for 4 days in FluorD3.

	Fluoride Solution	FluorD3
Sample size	105	105
Arithmetic mean	85.85	87.50
95% CI mean	82.74–88.96	84.28–90.72
Variance	257.95	277.21
Standard deviation	16.06	16.64
Standard error of the mean	1.56	1.62

**Table 2:** Summary statistics of formed crystals’ size after immersion in control and test solutions.

	Fluoride	FluorD3
Sample size	150	150
Arithmetic mean	1.49	1.54
95% CI mean	1.44–1.54	1.49–1.59
Variance	0.1	0.11
Standard deviation	0.31	0.32
Standard error of the mean	0.02	0.03

**Table 3:** Summary statistics of crystals’ number within the same 105 microscopic fields at T1 and T2.

	T1	T2
Sample size	105	105
Arithmetic mean	87.50	84.71
95% CI mean	84.28–90.72	81.53–87.89
Variance	277.21	270
Standard deviation	16.64	16.44
Standard error of the mean	1.62	1.60

**Table 4:** Summary statistics of each individual crystal size measured at T1 and T2.

	T1	T2
Sample size	15	15
Arithmetic mean	1.49	1.09
95% CI mean	1.22–1.77	0.88–1.30
Variance	0.24	0.14
Standard deviation	0.496	0.38
Standard error of the mean	0.12	0.09

**Table 5:** Summary statistics of each crystal size measured at T2 and T3.

	T2	T3
Sample size	15	15
Arithmetic mean	6.55	4.03
95% CI mean	5.76–7.34	3.57–4.49
Variance	2.03	0.69
Standard deviation	1.42	0.83
Standard error of the mean	0.36	0.21

## Data Availability

The data presented in this study are available on request from the corresponding author.
